# Effect of Physical Exercise Under Different Intensity and Antioxidative Supplementation for Plasma Superoxide Dismutase in Healthy Adults: Systematic Review and Network Meta-Analysis

**DOI:** 10.3389/fphys.2022.707176

**Published:** 2022-02-03

**Authors:** Yining Xu, Minjun Liang, Ukadike C. Ugbolue, Gusztáv Fekete, Yaodong Gu

**Affiliations:** ^1^Faculty of Sports Science, Ningbo University, Ningbo, China; ^2^School of Health & Life Sciences, University of the West of Scotland, South Lanarkshire, United Kingdom; ^3^Savaria Institute of Technology, Eötvös Loránd University, Szombathely, Hungary

**Keywords:** oxidant stress, physical exercise, superoxide dismutase, network meta-analysis, SOD, antioxidant supplements

## Abstract

**Background:**

The dynamic balance between oxidation and anti-oxidation in the body’s internal environment has a significant meaning for human health. Physical exercise and antioxidative supplementation could affect the balance of oxidation and anti-oxidation systems. The evidence on the effects of physical exercise and antioxidative supplementation is mixed.

**Aims:**

To identify the effects of physical exercise, antioxidative supplementation, and their combination on the dynamic balance between oxidation and anti-oxidation in different subgroups of healthy adults.

**Methods:**

All studies which reported randomized controlled trials with healthy participants were screened and included from the databases of PubMed, Medline, Embase, and Ovid. All participants were reclassified according to their different daily life activities. All physical exercise interventions were reclassified according to the intensity. The effect size would be calculated in percent or factor units from the mean level change with its associated random-effect variance.

**Result:**

There were 27 studies included in this review. The agreement between authors by using The Cochrane Collaboration Risk of Bias Assessment Tool reached a kappa-value of 0.72. Maintaining a regular physical exercise routine in an appropriate intensity would be beneficial to the body’s anti-oxidative potential. Anti-oxidative supplementation could have some positive but limited effects on the body’s anti-oxidative status and complex interaction with physical exercise.

**Conclusion:**

Keeping a regular physical exercise routine and gradually increasing its intensity according to the individual’s daily life activity might be a better choice to maintain and enhancing the body’s antioxidation potential, only using anti-oxidative supplementation is not recommended. More research is needed to explore the best combination protocol.

**Registration Number:**

CRD42021241995.

## Introduction

The dynamic balance between oxidation and anti-oxidation in the body’s internal environment has a significant meaning for human health. On the one hand, the body consumes oxygen through aerobic metabolism, providing the energy needed by the body, and producing oxidation by-products at the same time. On the other hand, the body reduces or removes oxidative by-products through a series of biochemical reactions, and ultimately achieves a dynamic balance between oxidation and antioxidation systems (Sies, 1991, 1997; Newsholme et al., 2016; Pizzino et al., 2017; Powers et al., 2020). At present, the academic community has limited consensus on this issue. First, the equilibrium point of this dynamic balance is still not clear, and people even could not determine the immediate direction of this dynamic balance from biochemical indicators. Moreover, due to the population difference, individual difference, and the difference of oxidative and anti-oxidative status of the same person under different conditions and ages, the normal values of the indicators that used to assess the dynamic balance between oxidation and antioxidation are still just referenced values, not the gold standard, in clinical practice.

Nevertheless, it is generally believed that the excessive tendency of this dynamic balance toward oxidation will cause negative effects on human health and has a close relationship with the pathogenesis of a variety of diseases (Taniyama and Griendling, 2003). The main mechanism might be that the by-products formed by oxidation reaction accumulate and could not be removed in time, which will cause damage to cells and molecules, and eventually form the so-called oxidative stress. Oxidative stress could cause health problems. For example, it is one of the main risk factors of myocardial infarction (Yan et al., 2018), and might also lead to liver damage (Rigamonti et al., 2003), diabetes, and cardiovascular disease (Lee et al., 2013; Newsholme et al., 2016). Meanwhile, oxidative stress is also related to the pathogenesis of hypertension (Briones and Touyz, 2010). Therefore, in reality, efforts have been made to induce this dynamic balance toward the direction of antioxidation.

For now, the methods commonly used to induce dynamic balance shift toward oxidation direction mainly come from two perspectives. One of the two perspectives aims to increase the body’s metabolic activity and accelerate the removal of oxidation by-products. Among all the methods from this perspective, physical exercise intervention is one of the commonly used methods (Fiogbé et al., 2017; Cuyul-Vásquez et al., 2020). However, when it comes to the effect of physical exercise on the dynamic balance between oxidation and antioxidation, the evidence shows a contradictory trend. Although physical exercise interventions have been proved to have a positive effect on the oxidation direction in many studies (Liu et al., 2005), there are still some studies that have found that physical exercise causes greater metabolic stress on the body, which could be detrimental to health. For example, a previous study demonstrated that short-term supramaximal anaerobic exercise induces oxidative stress (Groussard et al., 2003). The differences in evidence might come from the heterogeneity of the study protocols, particularly the different intensities of the physical exercise interventions, and the population of the participants in trials (Laitano et al., 2010; Knez et al., 2014). Previous studies found that the oxidative and anti-oxidative status of athletes related to their physical status (Dékány et al., 2006), there was no difference between the effect of moderate physical activity on oxidative stress indicators for premenopausal women and postmenopausal women (Hernández et al., 1999), and a 3-month-long specific training program improved the oxidation and anti-oxidation balance of elite karate athletes and could be recommended for athletes having similar physical fitness level (Jemili et al., 2017). Therefore, the academic community has not reached a consensus on the best physical exercise prescription for different populations, and the guidelines and statements of different countries, medical associations, and sports associations are different from each other.

The other one of the two perspectives is to increase the body’s antioxidative capacity by taking exogenous antioxidative supplementation. For example, a systematic review and meta-analysis claimed that the cocoa flavanols intake might reduce exercise-induced oxidative stress (Decroix et al., 2018), another systematic review concluded that curcumin might have a slight antioxidative effect (Fernández-Lázaro et al., 2020), a study whose participants were healthy male subjects found that Panax ginseng extract could elevate the activity of oxidation by-products scavenger enzymes such as catalase (CAT), and superoxide dismutase (SOD) (Kim et al., 2005), and groups controlled trial demonstrated that whey protein isolate could attenuate oxidative stress induced by intense exercise in trained cyclist men (Ton et al., 2017). But evidence on this perspective is still limited. Above all, due to the variety of antioxidative supplementation, different intake protocols, and different experimental backgrounds such as the population of the participants (Cui et al., 2004; Koenig et al., 2015; Heidarianpour and Yaganah, 2020), there is great heterogeneity among the studies of anti-oxidative supplementation. Second, there is a lack of high-quality human randomized controlled trials, a considerable part of the trials on antioxidative supplementation is still in the animal experiment stage (Notin et al., 2010; Lee et al., 2013; Koozehchian et al., 2018; Sharp et al., 2020).

Meanwhile, some researchers explore the effect of multi-model interventions such as a combination of physical exercise and antioxidative supplement intake. For example, a systematic review and meta-analysis concluded that vitamins C and E could be used to prevent oxidative stress, but could not enhance performance or improve muscle mass (Dutra et al., 2020), another randomized, double-blind, crossover study found that coenzyme Q10 supplementation could partially prevent the increase in lipid peroxidation after repeated short-term supramaximal exercise (Gül et al., 2011), a three-armed trial showed that regular physical activities and massage manipulations could significantly increase SOD activities (Karabulut et al., 2013), and a randomized controlled trial claimed that a combination of exercise training and hypoxic exposure could improve oxidation status of obese individuals (Verges et al., 2020). Similarly, because of the difference in intervention protocols, differences in measures, and the fact that some trials study acute effects and others long-term effects, it is difficult to synthesize the evidence.

Therefore, to discuss this issue, a systematic review of the available evidence is necessary. To increase the statistical consistency among the evidence, the review reclassified all the trials of included studies before synthesis the evidence. First of all, considering that the population differences might greatly affect the body’s capacity to adapt to the oxidative stress, all the participants of the trials of included studies were divided into four categories according to their different daily physical activity, which was physically inactive population, generally physically active population, physically active population, and professional athletes. At the same time, considering that the heterogeneity within the intervention protocols, the physical exercise intervention protocols used in the included studies were divided into different categories according to different intensities in this review.

The plasma superoxide dismutase (SOD) is the primary outcome measure of the meta-analysis. The plasma SOD, which is also called EC-SOD, is one of the main antioxidative enzymes out of the cell (Ji et al., 2016), and has been regarded as the first line of defense of antioxidative enzymes. The gene of EC-SOD was found to be the most differentially over-expressed in the brains of queen vs worker ants. This finding raises the possibility of an important role of antioxidant function in modulating lifespan (Lucas and Keller, 2018). The EC-SOD could catalyze O_2_^–^ anion dismutation into O_2_ and H_2_O_2_, avoid excessive accumulation to produce toxicity (Bresciani et al., 2015). It is demonstrated in the animal model that the absence of EC-SOD was found to be without effect on the lifespan of mice, however, it might lead to a higher risk of oxidation-induced damage such as premature death under high oxygen tension. Moreover, the extra-cellular superoxide dismutase (EC-SOD) would contribute to the development of hypertension (Gongora et al., 2006; Lob et al., 2010). Diminished SOD3 activity has been linked to lung diseases such as Acute Respiratory Distress Syndrome (ARDS) or Chronic obstructive pulmonary disease (COPD) (Young et al., 2006; Gongora et al., 2008; Ganguly et al., 2009). Plasma SOD could partly reflect the dynamic balance between oxidation and anti-oxidation of the body, and abrupt change of plasma SOD might indicate the destruction of the dynamic balance between oxidation and anti-oxidation of the body (McCord and Fridovich, 1969; Beauchamp and Fridovich, 1971). In this review, plasma SOD is selected as an indicator that represented the tilting trend of the body’s balance between oxidation and anti-oxidation to the direction of anti-oxidation. However, what should be emphasized is that the increase of plasma SOD could not be equal to the final change of oxidation and anti-oxidation balance, because the balance is a complex system with multiple indicators, and the assessment of the final direction of the balance should synthesize more clinical and laboratory data.

There are many methods for measuring plasma SOD, and different methods might use different equipment or instruments and have different standards. For example, the activity of SOD could be measured at 37°C and expressed in U/g Hb by using different commercially available kits (Lamprecht et al., 2008), it could also be measured through a colorimetric assay by using a commercially available colorimetric method and expressed in U/ml (Zini et al., 2002). Moreover, spectro-photometrically standard quantification methods with the unit of mol/L/min, or a microplate reader method with the unit of pkat/g Hb (McCord and Fridovich, 1969; Beauchamp and Fridovich, 1971; Aebi, 1984; Flohe and Gunzler, 1984; Chirico et al., 2012). Therefore, for the clinical application and increasing the consistency of the meta-analysis, all the data would be synthesized at the same scale in this review, the effect size would be calculated in percent or factor units from the mean level change and its associated random-effect variance.

## Methods

### Protocol and Registration

This systematic review was written according to the Preferred Reporting Items for Systematic Reviews and Meta-Analysis guidelines (PRISMA) (Moher et al., 2009). Literature eligibility criteria, exclusion criteria, and study search strategy were proposed and agreed upon by two independent authors (YX and ML) to minimize bias. The PROSPERO Registration Number of this systematic review was CRD42021241995.

### Eligibility Criteria

#### Population/Participants

All studies whose participants were healthy adults would be included in this review. Since the level of daily life activity might affect the individual’s capacity of anti-oxidation, all the participants would be reclassified into 4 groups, which were (1) inactive group, the adults in which were obese, sedentary, and over-weight, (2) general active group, the adults in which were elderly, middle-aged, untrained, and others that kept a normal daily physical activity, (3) active group, the adults in which kept a regular physical exercise but were not professional athletes, (4) athlete group, the adults in which were professional athletes, semi-professional athletes, and student-athletes in high schools or universities.

#### Intervention(s)

The included studies in this systematic review should contain randomized controlled trials which explore the effects of physical exercise and antioxidative supplementation. As had been explained in the introduction section, all the physical exercise interventions would be reclassified into (1) Low-intensity exercises (LIE), which referred to physical exercise under an intensity from 0 to 60% VO_2*max*_ or HR_*max*_, (2) Moderate-intensity exercise (MIE), which referred to physical exercise under an intensity from 60 to 80% VO_2*max*_ or HR_*max*_, (3) High-intensity exercise (HIE), which referred to physical exercise under an intensity more than 80% VO_2*max*_ or HR_*max*_ and High-Intensity Interval Training, (4) Resistance Training (RT), (5) Passive Physiotherapy (PT), (6) Maintaining current life or non-treatment intervention (Blank).

Moreover, all the interventions would be tagged by “Long-term (LT)” and “Acute,” the former of which referred to interventions that last more than one week and the latter referred to interventions that were conducted only once before the blood samples collection. The two dimensions of interventions reclassification would be present as acronyms connected by a blank. For example, a three-week high-intensity exercise would be presented as “LT HIE.”

What should be emphasized was that some specific training for a particular event would be reclassified according to the metabolic characteristics of the event itself. For example, a running Exercise at the intensity of 65% to 75% maximal heart rate would be reclassified into the acute moderate-intensity group (Acute MIE).

The capital letter “S” referred to that the participants took antioxidative supplementation during the trial. Similarly, if the participants took antioxidative supplementation for more than one week, they would be tagged by “Long-term supplement intake (LT S),” and if the participants took supplementation only once before the blood samples collection or exercise test, they would be tagged by “Acute S.” It should be emphasized that antioxidative supplement intake protocols in different doses would be treated as independently separated operate groups.

A plus sign would be used to represent a combination of supplementation and physical exercise intervention. For example, “LT S + LT HIE” meant that the participants of a long-term study whose intervention was a combination of antioxidative supplementation and high-intensity exercise throughout three weeks.

The result of intervention reclassification would be presented in the table of included studies’ information.

#### Comparator(s)

The Bayesian approach to network meta-analysis is particularly flexible for fitting complex models including multi-arm trials and provides credible intervals and rank probabilities for competing treatments to be the best, second best, and so on. The advantage of the Bayesian approach is that competing treatments could be ranked for overall effectiveness. All the treatments would be ranked by their estimated effect sizes, and then, across all samples, averages for the first rank, second rank, and so on (Lumley, 2002; Chung and Lumley, 2008; Salanti et al., 2011; Wang et al., 2012). These estimated probabilities are plotted against the ranks. Since the network meta-analysis is based upon the Bayes’ theorem (Mills et al., 2013), it is feasible to make mixed and indirect intervention comparisons. The eligibility criteria of the comparator(s) would be the same as the intervention(s).

#### Outcomes

Plasma SOD was chosen as the primary outcome measure of this systematic review. It should be emphasized that the superoxide dismutase of myocardial cells and muscle fibers would be excluded. And all the outcomes would be input into two different analyses, one of which was the change in plasma SOD at resting-state more than 48 h after exercise, the other was the change in plasma SOD immediately or within 48 h after exercise.

#### Study Design

Only studies of randomized controlled trials would be eligible for this systematic review.

#### Exclusion Criteria

Studies of trials that had the following characteristics would be excluded. (1) The participants of the trial were unhealthy adults, teenagers under 18 years old, and children. (2) The article didn’t provide the original data of plasma SOD. (3) The participants of the trial were from a mixed population. (4) The intervention of the trial couldn’t be reclassified into LIE, MIE, HIE, RT, and PT such as injection treatments. (5) The participants of the trial took prescription drugs as treatment. (6) The study was a published abstract.

### Information Sources

A comprehensive, reproducible search strategy was performed on the following databases from January 2000 to March 2021: PubMed, Embase, Medline, and Ovid. Reference lists of included studies were also searched. Gray literature was searched to identify potential studies. The authors would be contacted and requested for missing data when the data of articles were insufficient.

### Search Strategy

The search terms used in each database was as follows: (1) in PubMed, the search term was “{(redox) OR (oxidative) OR (oxidative) [Title/Abstract]} AND {(exercise) OR (train*) [Title]}”; (2) in Medline and Ovid, the search term was “(AB redox OR oxidative OR oxidative) AND (TI exercise OR train OR training) AND (AB randomized OR randomized) NOT (TI design or protocol or review)”; (3) in EMBASE, the search term was “(AB redox OR oxidative OR oxidative) AND (TI exercise OR train OR training) AND (AB randomized OR randomized).”

### Study Selection

All potential studies were imported into EndNote X9 (Thomson Reuters, Carlsbad, CA, United States) and duplicates would be removed. Title, abstract, and full-text screening were made by two independent authors (YX and ML). Any disagreement would be resolved by a third independent reviewer (UU).

### Data Collection Process

Data were extracted by two independent authors (YX and ML). Any discrepancies would be solved by an independent arbitrator (UU).

### Data Items

The following information would be collected and recorded. (1) Demographic characteristics, such as mean age, gender, body mass index (BMI), and body fat mass percentage. (2) Details of the trial design, such as sample size, intervention protocol, and follow-up times (if the participants took antioxidative supplementation, the course of intake would also be recorded). (3) Data of outcome measure in baseline and each follow-up time.

### Risk of Bias Assessment

The Cochrane Collaboration Risk of Bias Assessment Tool was used to make the risk of bias assessment. All the included studies were assessed by two independent authors (YX and ML). Any disagreement would be discussed, and an independent arbitrator (UU) was invited when an agreement could not be met. Agreement between authors was determined by Cohen’s Kappa value.

### Summary Measures

Data pre-processing and analysis were made by two independent investigators (YX and ML). Microsoft Office Excel (Version 16.0 Microsoft Corporation, Redmond, WA, United States) was used to pre-process the original data, transferring all the outcomes to the form of mean value and its standard deviation (Mean ± SD).

### Synthesis of Results

The Aggregate Data Drug Information System (ADDIS V1.16.8 Produced by Drugis.org^1^) was used to analyze all the processed data, calculated effect size, pool data into network meta-analysis, and output all the results and figures. The effect size would be calculated in percent or factor units from the mean level change with its associated random-effect variance and expressed in the form of Mean Difference% ± SD%.

### Risk of Bias Across Studies

The Cochrane Collaboration Risk of Bias Assessment Tool was used to assessing the risk of bias across the included studies (Robertson et al., 2014). The assessment would be conducted according to the results of the bias risk assessment of individual studies.

### Network Meta-Analysis

#### Network Geometry

The network geometries displayed the overall number and type of interventions by the Bayesian simulation modeling and provided key information about the strength of evidence. In the network geometry, every node represented one of the interventions, while the lines referred to the available direct comparisons between each pair of interventions, and the amount of available information could be presented by “weight” the edge using numbers of arms on them(Salanti et al., 2011).

#### Consistency and Inconsistency Analysis

Network meta-analysis gives a consistent, integrated picture of the relative effects. However, given such a consistent set of relative effect estimates, it may still be difficult to conclude a potentially large set of treatments. Fortunately, the Bayesian approach makes it possible to process complex data, to estimate the probability that is given by the priors and the data. Moreover, the researcher is allowed and even encouraged to use all available information, in particular prior information that is not generated by the data. The Bayesian model framework also requires “prior information” in the form of distribution around the parameters of interest. The results would be shown in the rank probability plot. The sum of all rank probabilities is 1, both within a rank over treatments and within a treatment over ranks (Dias et al., 2013).

If there are closed loops in the evidence structure, the network meta-analysis could be called a mixed intervention comparison, and the inconsistency of the evidence should be assessed because in network meta-analysis the evidence structure is more complex. Inconsistency assessment could occur when an intervention C has a different effect when it is compared with A or B. Inconsistency may even occur with normal Meta-analysis, however, it could only be detected using a network meta-analysis. If there is no relevant inconsistency, or there is no closed loop in the evidence structure, the network meta-analysis could be called an adjusted indirect intervention comparison, and a consistency model could be used to conclude the relative effect of the included interventions (Lu and Ades, 2006).

The valid results of network meta-analysis depended on the evidence network being internally consistent: direct and various sources of indirect evidence should be in agreement. Inconsistency referred to differences between direct and indirect effect estimates for the same comparison and significant inconsistency threatened the validity of the results of a network meta-analysis. Therefore, if inconsistency occurred, further exploration of inconsistency would be needed to identify possible sources of disagreement. The random-effects standard deviations calculated under both consistency and inconsistency models could be used to identify if there was inconsistency within interventions. If random effects standard deviations calculated under both consistency and inconsistency models were fully identical, it meant that there was a good consistency with the interventions. If not, the *P*-values from the analysis of the node splitting would be checked to determine which model would be used (Dias et al., 2010).

The node-splitting analysis is an alternative method to assess inconsistency in network meta-analysis when the results are easier to interpret. The node-splitting analysis requires a separate model to be run for each node to be split and assesses whether direct and indirect evidence on a specific node (the split node) are in agreement to identify consistency discrepancies that are associated with specific nodes. The node-splitting analysis is performed within a Bayesian framework and is computationally more intensive than other approaches. Whether the identified discrepancy is statistically significant could be determined by examining the calculating a respective Bayesian *P*-value.

#### Ranking of Measures and Probability

A ranking of measures and probability would be made to facilitate simultaneous inference regarding interventions. A table showing the ranking of treatments would be made according to the probability of each intervention being the most effective or the least effective. The overall sum of the percentage in each row or column should be 1.00 (100%). Probabilities are estimated for an intervention to be ranked at a specific place (first, second, and so on) according to each outcome. However, the probability of being the best does not account for the uncertainty in the relative intervention effects and can spuriously give higher ranks to interventions for which little evidence is available. The probability of being the best has the disadvantage that it does not reflect the spread of rankings for the treatments and to consider just the crude figures may be misleading. Therefore, a ranking of interventions based solely on the probability of each intervention being the best should be avoided (Salanti et al., 2011).

### Additional Analysis

#### Pair-Wise Meta-Analysis

If a comparison of two interventions was appearing separately, a pair-wise meta-analysis should be made. The effect size would be calculated in percent or factor units from the mean level change with its associated random-effect variance and expressed in the form of Mean Difference% ± SD%. The result would be presented by a forest plot and the heterogeneity within studies would be assessed according to the statistic I^2^. The software Review Manager (Version 5.3, The Cochrane Collaboration, St. Albans House, 57-59 Haymarket, London, United Kingdom) would be used to make the pair-wise meta-analysis.

#### Meta-Regression

Regression, or multiple regression, is used to assess the relationship between one or more covariates and a dependent variable. The same approach could be used with meta-analysis. Meta-regression refers to these procedures when they are used in a meta-analysis. One of the differences is that it should assign a weight to each study and the need to select the appropriate model (fixed versus random effects), the other is a meta-regression would be meaningful only with more than 10 trials included. Also, as was true for subgroup analyses, the R^2^ index, which is used to quantify the proportion of variance explained by the covariates, must be modified for use in the meta-analysis (Higgins, 2009). The software STATA^®^ (Version 12, StataCorp LLC, College Station, TX, United States) would be used to make the meta-regression.

## Results

### Search Strategy and Information Extraction

**FIGURE 1:**
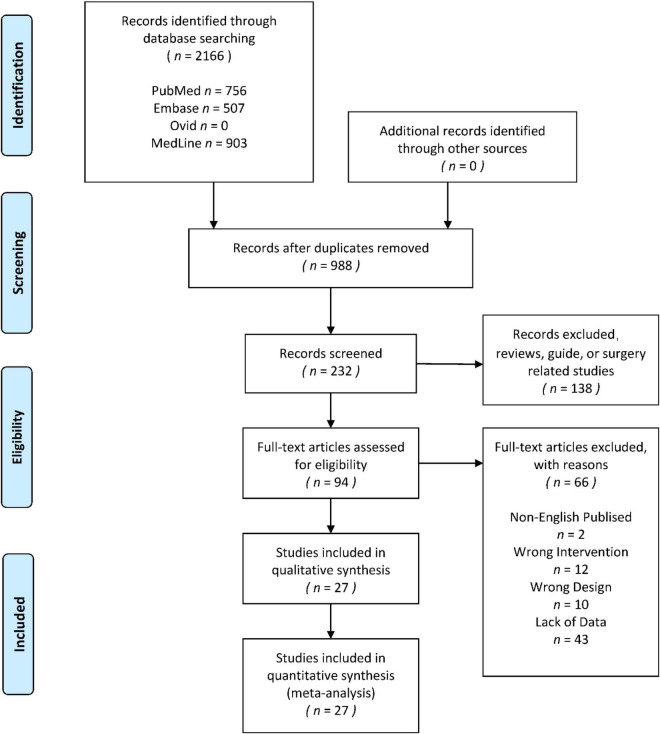
Preferred Reporting Items for Systematic Reviews and Meta-Analysis guidelines (PRISMA) flow diagram for the systematic review and network meta-analysis.

The search yielded 2166 titles and abstracts for screening. Eventually, 27 studies (Margaritis et al., 2003; Pilaczynska-Szczesniak et al., 2005; Lu et al., 2006; Tauler et al., 2006; Wang et al., 2006; Sureda et al., 2008; Kim et al., 2010; De Marchi et al., 2012; Jówko et al., 2012, 2015; Savory et al., 2012; Tartibian and Maleki, 2012; Deminice et al., 2013; Mila-Kierzenkowska et al., 2013; da Silva et al., 2014; Mazani et al., 2014; Carrera-Quintanar et al., 2015; Levers et al., 2015; Bunpo and Anthony, 2016; Friedenreich et al., 2016; Hajizadeh Maleki et al., 2017; Duranti et al., 2018; Giolo et al., 2018; Yimcharoen et al., 2019; Chacaroun et al., 2020; de Castro et al., 2020; Dani et al., 2021) were included in the final analysis. The 27 studies could be divided into 39 independent randomized controlled trials, and 17 of the 39 trials reported the long-term effect of the interventions, 22 reported the acute effect of the interventions, and 5 trials reported both long-term effect and acute effect. The identification process is shown by a flow diagram (Moher et al., 2009), which is in Figure 1. The information of all included studies would be shown in Tables 1, 2. All the original data was provided in the Supplementary File.

**TABLE 1:** Information of the included studies explored the effect of different supplementation and physical exercise protocols on the increasing plasma superoxide dismutase (SOD) after exercise tests.

Study	Participants	Population	Age (years)	BMI (kg/m^2^)	Fat (%)	Intervention	Classification
De Marchi et al. (2012)	Untrained Male	Inactive	23			Low-Level Laser Therapy	Acute PT
						Placebo Therapy	Acute Placebo
Wang et al. (2006)	Healthy Sedentary Men	Inactive	24	22.88		0–40% VO_2*max*_ Exhausting Pedaling	Acute LIE
						60% VO_2*max*_ Exhausting Pedaling	Acute MIE
						80% VO_2*max*_ Exhausting Pedaling	Acute HIE
de Castro et al. (2020)	Elderly Women	Generally active	67	27.63		High-velocity RT @70% 10RM	Acute RT
						Traditional RT	Acute RT
Kim et al. (2010)	Fight Pilots	Active	22	24.07	17.83	Weight Training	Acute RT
						Running Training	Acute HIE
						Orbotron Training	Acute HIE
	Fight Pilots	Active	22	24.07	17.83	9 weeks Weighted Training	LT RT
						9 weeks Running Training	LT HIE
						9 weeks Orbotron Training	LT HIE
Mila-Kierzenkowska et al. (2013)	Professional Male Volleyball Player	Athlete	28	23.63		Whole-body cryotherapy + 40min Submaximal Exercise	Acute PT + Acute HIE
						Normal Exercise @85%HRmax	Acute HIE
Savory et al. (2012)	Over-Weight People	Inactive	34	25.4		3 weeks Selenium Supplementation + Incremental maximal exercise test @40–70% VO_2*max*_	LT S + Acute HIE
						3 weeks Placebo + Incremental maximal exercise test @40–70% VO2max	LT Placebo + Acute HIE
	Normal-Weight People	Generally active	34	25.4		3 weeks Selenium Supplementation + Incremental maximal exercise test @40–70% VO2max	LT S + Acute HIE
						3 weeks Placebo + Incremental maximal exercise test @40–70% VO_2*max*_	LT Placebo + Acute HIE
da Silva et al. (2014)	Young Male Students	Generally active	21	25.25		2 weeks Taurine + Exhausting RT	LT S + Acute RT
						2 weeks Placebo + Exhausting RT	LT Placebo + Acute RT
Duranti et al. (2018)	Healthy Young Men	Generally active	26	23.4		2 weeks Quercetin + Eccentric RT	LT S + Acute RT
						2 weeks Placebo + Eccentric RT	LT Placebo + Acute RT
Yimcharoen et al. (2019)	Healthy women	Generally active	22	21.2		Ascorbic Acid + 30min Moderate-Intensity Cycling	Acute S + Acute MIE
						Placebo + 30min Moderate-Intensity Cycling	Acute Placebo + Acute MIE
Lu et al. (2006)	Students of National College of Physical Education	Active	22			3 weeks Spirulina Platensis + Exhausting Treadmill Exercise	LT S + Acute HIE
						3 weeks Placebo + Exhausting Treadmill Exercise	LT Placebo + Acute HIE
Levers et al. (2015)	Resistance Trained Males	Active	21	26.07	14.2	10 days Powdered Montmorency Tart Cherry Supplementation + 10 × 10 Squat @70% 1RM	LT S + Acute RT
						10 days Placebo + 10 × 10 Squat @70% 1RM	LT Placebo + Acute RT
Sureda et al. (2008)	Male amateur trained runners	Active	35	23.1		1-month Vitamin E and Vitamin C + Half-Marathon Race	LT S + Acute MIE
						10 days Placebo + 10*10 Squat @70% 1RM	LT Placebo Intake + Acute RT
Tartibian and Maleki (2012)	Healthy non-professional male road cyclists	Active	24	21.99	8.05	2 weeks Honey + Intense cycling training	LT S + LT HIE
						Placebo + 2 weeks Intense cycling training	LT Placebo + LT HIE
	Healthy non-professional male road cyclists	Active	24	21.99	8.05	8 weeks Honey + Intense cycling training	LT S + LT HIE
						Placebo + 2 weeks Intense cycling training	LT Placebo + LT HIE
Mazani et al. (2014)	Healthy Young Males Athletes	Athlete	19	22.98		2 weeks Pomegranate + Exhausting Exercise @70% HRmax on Treadmill	LT S + Acute MIE
						2 weeks Placebo + Exhausting Exercise @70% HRmax on Treadmill	LT Placebo + Acute MIE
Tauler et al. (2006)	Amateur Trained male endurance athletes	Active	23	24.5		90 days Multi-Supplementation + Maximal and Submaximal Exercise Test	LT S + Acute HIE
						90 days Placebo + Maximal and Submaximal Exercise Test	LT Placebo Acute HIE
Deminice et al. (2013)	25 Soccer Players	Athlete	17	22.66	15.07	7 days Creatine + Running-based Anaerobic Sprint Tests	LT S + Acute HIE
						7 days Placebo + Running-based Anaerobic Sprint Tests	LT Placebo + Acute HIE
Jówko et al. (2015)	Male Sprinters	Athlete	22	23.5	11.8	4 weeks Green Tea Extract + Repeated Cycle Sprint Test	LT S + Acute HIE
						4 weeks Placebo + Repeated Cycle Sprint Test	LT Placebo + Acute HIE
Jówko et al. (2012)	Soccer Players	Athlete	23	23.6		Green Tea Polyphenols + Intense Muscle Endurance Test@60% 1RM	Acute S + Acute RT
						Placebo + Intense Muscle Endurance Test@60% 1RM	Acute Placebo + Acute RT
Pilaczynska-Szczesniak et al. (2005)	Rowing Athletes	Athlete	22	23.11		Incremental Rowing Exercise	Acute HIE
						1-month Chokeberry Juice + Incremental Rowing Exercise	LT S + Acute HIE
						1-month Placebo + Incremental Rowing Exercise	LT Placebo + Acute HIE

*LT, long-term; S, supplementation; LIE, low-intensity exercise; MIE, moderate-intensity exercise; HIE, high-intensity exercise; RT, resistance training.*

**TABLE 2:** Information of the included studies explored the effect of different physical exercise and supplementation protocols on increasing plasma superoxide dismutase (SOD) under resting state.

Study	Participants	Population	Age (years)	BMI (kg/m^2^)	Fat (%)	Intervention	Classification
Chacaroun et al. (2020)	Obese Individuals	Inactive	54	31.5	35.2	75% HRmax Cycling + Gas-mixing face mask	LT-HIE
						75% HRmax Cycling	LT-MIE
Friedenreich et al. (2016)	Healthy Postmenopausal Women	Generally active	61	29.15	42.3	A Year-Long Aerobic Exercise From 50–60% HRR to70–80% HRR	LT AE
						Maintain Current Life	Blank
da Silva et al. (2014)	Young Male Students	Generally active	21	25.25		Taurine	LT S
						Placebo	LT Placebo
Duranti et al. (2018)	Healthy Young Men	Generally active	26	23.4		Quercetin	LT S
						Placebo	LT Placebo
Hajizadeh Maleki et al. (2017)	Healthy Male	Generally active	31	27.03	22.28	Moderated-Intensity Continuous Training@Treadmill	MIE
						High-Intensity Continuous Training@Treadmill	HIE
						High-Intensity Interval Training@Treadmill	HIE
						Non-Exercise	Blank
Savory et al. (2012)	Over-Weight People	Inactive	34	25.4		Selenium Supplementation	LT S
						Placebo	LT Placebo
	Normal-Weight People	Generally active	34	25.4		Selenium Supplementation	LT S
						Placebo	LT Placebo
Bunpo and Anthony (2016)	Sedentary Adults	Inactive	24			Remain Sedentary	Blank
						Running Exercise @65-75%HRmax	LT MIE
						Running Exercise @65-75%HRmax + 250 mg Ascorbic Acid Supplementation	LT S + LT MIE
	Sedentary Adults	Inactive	24			Remain Sedentary	Blank
						Running Exercise @65-75%HRmax	LT MIE
						Running Exercise @65-75%HRmax + 500 mg Ascorbic Acid Supplementation	LT S + LT MIE
Dani et al. (2021)	Healthy Elderly Women	Generally active	59	27.11		Grape Juice	LT S
						Grape Juice + Multi-Exercise @7-8 Borg CR10 Scale	LT S + LT HIE
						Placebo + Multi-Exercise @7-8 Borg CR10 Scale	LT Placebo + LT HIE
Giolo et al. (2018)	Healthy and non-obese postmenopausal women	Generally active	54	26.63	35.08	Isoflavone + Aerobic Exercise and Resistance Training	LT S + LT AE + LT RT
						Placebo + Aerobic Exercise and Resistance Training	LT Placebo + LT AE + LT RT
Levers et al. (2015)	Resistance Trained Males	Active	21	26.07	14.2	Powdered Montmorency Tart Cherry Supplementation	LT S
						Placebo	LT Placebo
Margaritis et al. (2003)	French Male Tri-athletes	Athletes	33	22.09	11.46	Antioxidant + Multi-Intense Training	LT S + LT MIE
						Placebo + Multi-Intense Training	LT Placebo + LT MIE
Carrera-Quintanar et al. (2015)	Students of Sport Science and Physical Activity	Generally active	21	22.52	12.84	Placebo + 60 min Eccentric Contraction-based Resistance Training	LT Placebo + LT RT
						Lippia Extract + 60 min Eccentric Contraction-based Resistance Training	LT S + LT RT
	Students of Sport Science and Physical Activity	Generally active	21	22.52	12.84	Placebo + 60 min Eccentric Contraction-based Resistance Training	LT Placebo + LT RT
						Vitamin E and Vitamin C + 60 min Eccentric Contraction-based Resistance Training	LT S + LT RT
	Students of Sport Science and Physical Activity	Generally active	21	22.52	12.84	Placebo + 60 min Eccentric Contraction-based Resistance Training	LT Placebo + LT RT
						Vitamin E and Vitamin C + 60 min Eccentric Contraction-based Resistance Training	LT S + LT RT
Mazani et al. (2014)	Healthy Young Males Athletes	Athletes	19	22.98		Pomegranate	LT S
						Placebo	LT Placebo

*LT, long-term; S, supplementation; LIE, low-intensity exercise; MIE, moderate-intensity exercise; HIE, high-intensity exercise; RT, resistance training.*

### Risk of Bias Assessment

**FIGURE 2:**
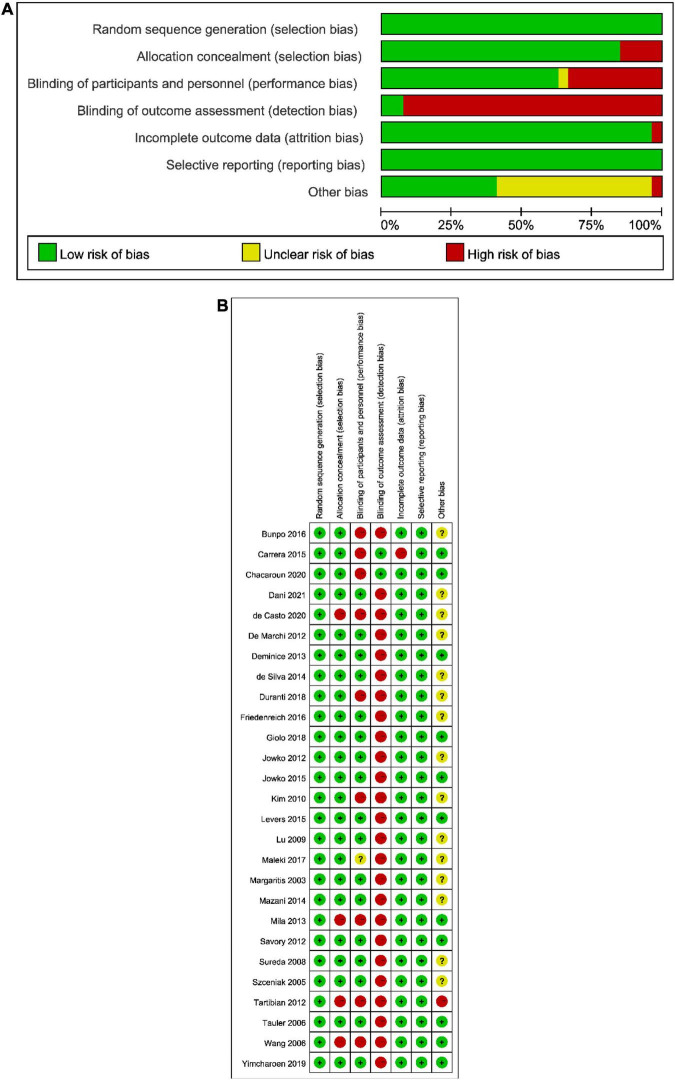
The result of the risk of bias assessment. **(A)** Risk of bias graph; **(B)** Risk of bias summary.

The risk of bias assessment is provided in Figure 2. A consensus was obtained for all items after discussion. The overall bias could be seen in Figure 2A: (1) the risk of performance bias (blinding of participants and personnel) was moderate (high in 9 studies); (2) the risk of detection bias (blinding of outcome assessors) was high (high in 21 studies); (3) the risk of attribution bias (incomplete outcome data) was low (low in 21 studies); (4) the risk of selection bias (random sequence generation and allocation concealment) was low (low in all studies); (5) the risk of reporting bias (selective reporting of outcomes) was low (low in all studies). According to Figure 2B, which showed the overall results, 4 studies had a high risk of bias, 5 studies had a moderate risk of bias, and 18 studies had a low risk of bias. The agreement between authors reached a kappa-value of 0.72.

### Meta-Analysis

#### Resting-State Effect

The network geometry of different physical exercise and supplementation protocols for increasing plasma SOD under resting-state, in each geometry, every node represented one of the competing interventions, while the lines corresponded to the available direct comparisons between each pair of interventions, and the amount of available information could be presented numbers of arms on the edges. For the physically inactive population, there were 1 mixed interventions comparison and 1 direct comparison, for the generally physically active population, there were 2 mixed intervention comparisons and 2 direct comparisons, for the physically active population, there was only 1 direct comparison, and for professional athletes, there were 2 direct comparisons. The network geometry would be provided in the Supplementary File.

Table 3 showed the random-effects standard deviation with its 95% confident intervals of each mixed interventions comparison under consistency model and inconsistency model. Since all the random-effects standard deviation with its 95% confident intervals under the inconsistency model were almost the same as those under the consistency models, the consistency model should be used.

**TABLE 3:** The random-effects standard deviation of mixed interventions comparisons under consistency and inconsistency models.

Population	Number of trials	Comparison	Random-effects standard deviation Mean(95% CI)	
			Consistency	Inconsistency
Physically inactive	7	Blank, LT HIE, LT S + LT MIE, LT MIE	1.73 (0.07, 20.65)	1.73 (0.04, 24.55)
Generally physically active	4	Blank, LT HIE, LT MIE, LT LIE	2.96 (0.09, 5.69)	2.93 (0.20, 5.69)
	6	LT S + LT HIE, LT Placebo + LT HIE, LT S, LT Placebo	19.52 (5.60, 58.65)	19.79 (5.60, 58.22)

*LT, long-term; S, supplementation; LIE, low-intensity exercise; MIE, moderate-intensity exercise; HIE, high-intensity exercise; RT, resistance training.*

The ranking of measures and probabilities would be presented in Table 4. Since the larger increase of plasma SOD indicated a higher anti-oxidative level, Rank 1 meant the best one whereas rank N meant the worst one. It could be seen that, in the mixed comparison of long-term high-intensity exercise, long-term moderate-intensity exercise with anti-oxidative supplementation, long-term moderate-intensity exercise without anti-oxidative supplementation, and maintaining current life, maintaining current life might be better than others for individual’s plasma SOD level. Moreover, for generally physically active individuals, long-term moderate-intensity exercise might be better than long-term high-intensity exercise, long-term low-intensity exercise, and maintaining current life for the body’s plasma SOD level, whereas a long-term combination of anti-oxidative supplementation and high-intensity exercise might be better than long-term protocols of anti-oxidative supplementation and placebo intake.

**TABLE 4:** The ranking of measures and probabilities of interventions’ effect on increasing plasma superoxide dismutase (SOD) under resting-state.

Population	Number of trials	Intervention	Rank 1	Rank 2	Rank 3	Rank 4
Physically inactive	7	Blank	0.76	0.22	0.02	0.00
		LT HIE	0.01	0.01	0.03	0.96
		LT S + LT MIE	0.01	0.02	0.93	0.04
		LT MIE	0.22	0.75	0.02	0.00
Generally physically active	4	Blank	0.08	0.24	0.64	0.04
		LT HIE	0.33	0.44	0.15	0.08
		LT LIE	0.01	0.04	0.11	0.84
		LT MIE	0.57	0.29	0.10	0.04
	6	LT S + LT HIE	0.72	0.08	0.18	0.02
		LT Placebo + LT HIE	0.02	0.16	0.09	0.74
		LT S	0.15	0.47	0.32	0.06
		LT Placebo	0.11	0.29	0.42	0.18

*LT, long-term; S, supplementation; LIE, low-intensity exercise; MIE, moderate-intensity exercise; HIE, high-intensity exercise; RT, resistance training.*

**FIGURE 3:**
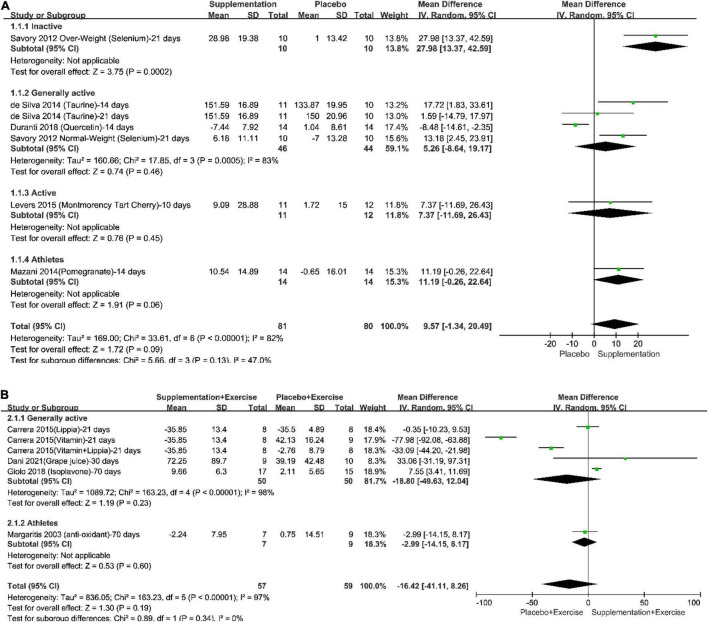
Forest plots of the pooled effect of different supplementation protocols on the increasing plasma superoxide dismutase (SOD) under resting-state. **(A)** Long-term supplementation vs. Long-term placebo intake; **(B)** Long-term combination of supplementation and physical exercise vs. Long-term combination of placebo intake and physical exercise.

The pooled effect of different supplementation protocols on increasing plasma SOD under resting-state could be seen in the forest plots, as in Figure 3. The results of meta-analyses in both total population (Total in the Figure) and subgroups of the population were provided. According to the results and the included studies, it could be seen that (1) a long-term supplementation could increase plasma SOD by 9.57% with a 95% confident interval from −1.34% to 20.49% and a *P*-value which was 0.09; (2) the effect of long-term supplementation on increasing plasma SOD was statistically significant positive, showing that a 3-week selenium supplementation protocol could increase plasma SOD by 27.98% (*p* < 0.05), however, there was only 1 trial recruited physically inactive participants; (3) None of the pooled effects in generally physically active population, physically active population, and professional athletes reached statistical significance, however, all of their results tend to support anti-oxidative supplementation; (4) The total heterogeneity was 82% and the difference within subgroups was 47%; (5) the trials whose interventions were long-term combination of supplementation and physical exercise only recruited generally physically active subjects and professional athletes, and the pooled effect showed that long-term combination of supplementation and physical exercise might not be better than placebo intake on the level of plasma SOD.

#### Acute Effect After Physical Exercise

The network geometry of the interventions for plasma SOD after exercise tests under different intensities, in each geometry, every node represented one of the competing interventions, while the lines corresponded to the available direct comparisons between each pair of interventions, and the amount of available information could be presented numbers of arms on the edges. According to the network geometry, for physically inactive population, there were 1 mixed intervention comparison and 2 direct comparisons, for generally physically active population, there were 3 direct comparisons, for physically active population, there were 6 direct comparisons, and for professional athletes, there were 2 direct comparisons and 1 mixed intervention comparison. The network geometry would be provided in the Supplementary File.

Table 5 showed the random-effects standard deviation with its 95% confident intervals of each mixed interventions comparison under consistency model and inconsistency model. Since all the random-effects standard deviation with its 95% confident intervals under the inconsistency model were almost the same as those under the consistency models, the consistency model should be used.

**TABLE 5:** The random-effects standard deviation of mixed interventions comparisons under consistency and inconsistency models.

Population	Number of trials	Comparison	Random-effects standard deviation Mean(95% CI)	
			Consistency	Inconsistency
Physically inactive	3	Acute HIE, Acute LIE, Acute MIE	5.79 (0.37, 11.06)	5.77 (0.35, 11.04)
Professional athletes	6	Acute HIE, LT S + Acute HIE, LT Placebo + Acute HIE, Acute HIE + Acute PT	10.49 (3.82, 15.73)	10.58 (4.32, 15.71)

*LT, long-term; S, supplementation; LIE, low-intensity exercise; MIE, moderate-intensity exercise; HIE, high-intensity exercise; RT, resistance training.*

The ranking of measures and probabilities would be presented in Table 6. Since the larger increase of plasma SOD indicated a higher anti-oxidative level, Rank 1 meant the best one whereas rank N meant the worst one. It could be seen that, for generally inactive individuals, high-intensity exercise test and moderate-intensity exercise test might elevate an individual’s plasma SOD level more than low-intensity exercise test. Moreover, a similar phenomenon also happened to professional athletes, a high-intensity exercise test or a combination of high-intensity exercise tests and physical therapy might elevate an individual’s plasma SOD level more than a high-intensity exercise test after a single dose intake of anti-oxidative supplementation.

**TABLE 6:** The ranking of measures and probabilities of interventions’ effect on the acute increase of plasma superoxide dismutase (SOD).

Population	Number of trials	Intervention	Rank 1	Rank 2	Rank 3	Rank 4
Physically inactive	3	Acute HIE	0.47	0.48	0.04	
		Acute LIE	0.02	0.07	0.91	
		Acute MIE	0.51	0.45	0.04	
Professional athletes	6	Acute HIE	0.36	0.38	0.20	0.06
		LT S + Acute HIE	0.04	0.13	0.26	0.57
		LT Placebo + Acute HIE	0.30	0.27	0.35	0.08
		Acute HIE + Acute PT	0.30	0.22	0.19	0.29

*LT, long-term; S, supplementation; LIE, low-intensity exercise; MIE, moderate-intensity exercise; HIE, high-intensity exercise; RT, resistance training.*

**FIGURE 4:**
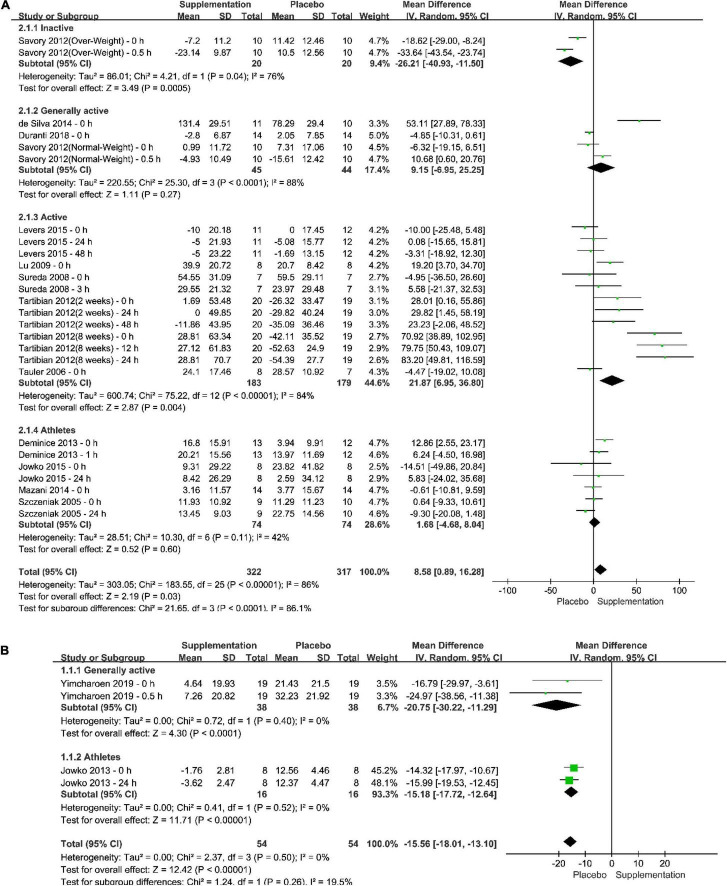
Forest plots of the pooled effect of different supplementation protocols on the increasing plasma superoxide dismutase (SOD) after exercise tests. **(A)** Long-term supplementation vs. Long-term placebo intake; **(B)** One-time supplementation vs. One-time placebo intake.

The pooled effect of different supplementation protocols on the increasing plasma SOD after exercise tests could be seen in the forest plots, as in Figure 4. The results of meta-analyses in both total population (Total in the Figure) and subgroups of the population were provided. According to the results and the included studies, it could be seen that (1) a long-term supplementation could increase plasma SOD after exercise test significantly by 8.58% with a 95% confident interval from 0.89% to 16.28% and a *P*-value which was less than 0.05; (2) the pooled effect in physically inactive population was significantly negative, whereas that in other populations were positive, especially in physically active population, the pooled effect showed that a long-term anti-oxidative supplementation could elevated plasma SOD level after an exercise test significantly by 21.87% with a *P*-value less than 0.05; (3) it seemed that, to some extent, the effect of long-term anti-oxidative supplementation depends on the duration of intake, a longer duration of intake might be better than a short-term of intake; (4) The total heterogeneity was 86% and the difference within subgroups was 86.1%; (5) only four trials reported the post-exercise test effect of one-time anti-oxidative supplementation in generally physically active subjects and professional athletes, and the pooled effect showed that one-time anti-oxidative supplementation might not be positive or better than placebo intake for plasma SOD level.

#### Meta-Regression

The result of meta-regression between populations in different levels of daily life physical activity and the increase of plasma SOD is shown in Table 7. The *k*-value is the number of included trials. According to the results, the *P*-value of Knapp-Hartung modification of the anti-oxidative supplementation and plasma SOD after physical exercise test was less than 0.05, and the covariates could explain 26.02% of the between-study variance. Moreover, the effect of anti-oxidative supplementation in physically inactive individuals, physically active individuals, and professional athletes were −26.26, 21.15, and −3.31 times as much as that in generally physically active individuals, seemed to show an inverted U-shaped trend.

**TABLE 7:** The result of Meta-regression between the different populations and the increase of plasma superoxide dismutase (SOD).

State	Collinearity	*k*	Covariates	Coefficient	t	*P*>|*t*|	Sig.	Adjusted *R*-squared
Resting-State	Physically active	13	Physically inactive	20.61	0.48	0.640	0.691	−14.19%
			Generally active	−14.96	−0.47	0.652		
			Professional athletes	7.37	−0.09	0.931		
After exercise test	Generally active	30	Physically inactive	−26.26	−1.38	0.180	0.035	26.02%
			Physically active	21.15	1.75	0.092		
			Professional athletes	−3.31	−0.26	0.795		

## Discussion

This systematic review and network meta-analysis might be able to help answer an important question, which is that exercise and anti-oxidative supplementation is friend or foe for the management of oxidative stress in healthy adults. As mentioned in the introduction section, the discussion of this review would be based on the following hypotheses: (1) The increase of plasma SOD indicates that the oxidation and antioxidant balance is moving to the direction of anti-oxidation to some extent; (2) Maintaining the oxidation and anti-oxidation balance in a state that favors anti-oxidation would be beneficial to the overall health of the body. Therefore, the increase of plasma SOD would be regarded as a positive result in this review. According to the result of meta-analyses, the primary findings of this systematic review are as follows.

First, for the oxidative and anti-oxidative balance under resting-state, maintain current life might be better than a long-term protocol of physical exercise under any intensity for physically inactive individuals’ plasma SOD level. Moreover, anti-oxidative supplementation seemed unable to change the results, or at least no better than maintaining current life. The reason might be that the oxidation and anti-oxidation balance of the physically inactive individuals has moved too much to the oxidative direction due to some healthy-negative factors such as sedentary, obesity, old age, etc., so that general anti-oxidative supplementation or daily physical exercise could no longer make the balance back to the anti-oxidative direction. This assumption has been confirmed by several studies. A systematic review with meta-analyses in 2018 showed that there remains uncertainty about the effect of exercise on oxidative stress in people with Down syndrome (Shields et al., 2018), a randomized clinical trial in 2016 reported that a year-long aerobic exercise intervention did not have a significant impact on oxidative stress in healthy but postmenopausal women (Friedenreich et al., 2016), and Fatouros’ team found that the benefits in anti-oxidation from endurance training might be reversed by training cessation (Fatouros et al., 2004). However, when it comes to generally physically active individuals, long-term high-intensity exercise might be more beneficial to the anti-oxidation capacity of the body and the effect might be amplified after a long-term anti-oxidative supplementation. A randomized clinical trial made by Wu’s team in 2017 found that high-intensity interval training could improve mitochondrial function and suppresses thrombin generation in platelets undergoing hypoxic stress (Wu et al., 2017), and a preliminary study in 2019 suggested that high-intensity exercise might lead to alterations in anti-oxidation and consequently to the physiological processes related to redox (Souza et al., 2019). Therefore, it could be speculated that, for improving the antioxidation capacity of the body through physical exercise, the intensity might be set according to the current daily life activity of an individual. People with lower daily life activity might need to start at a relatively lower intensity or increase their daily life activity at first. This speculation is consistent with the “Step by step” principle that is promoted by general exercise and health guidelines (Siedler et al., 2020).

Second, when discussing acute changes after exercise tests under different intensities, it could be found that a one-time moderate-intensity exercise test has the most possibility to increase more plasma SOD in physically inactive individuals, whereas for a professional athlete, a one-time high-intensity exercise test might be able to increase the level of plasma SOD to a large extent no matter whether this athlete intake anti-oxidative supplements or not. It might be speculated that, on one hand, a one-time physical exercise could stimulate the level of the body’s antioxidation to rise, and the extent of the rise might be related to the intensity of exercise and the individual’s daily life activity. On the other hand, the effect of a long-term anti-oxidative supplementation might not be as good as expected. And the speculated reason might be that, for professional athletes, who are already in adaptation to high oxidative stress caused by the high load of training volume, one time of intense training would stimulate their bodies to elevate their antioxidation levels, while a long-term anti-oxidative supplementation might have little or no extra value. Some previous studies have verified this hypothesis. As the results of Mazani’s study in 2014 (Mazani et al., 2014), a 2-week Pomegranate supplement intake might not be better than a 2-week placebo intake on increasing plasma SOD under resting-state for professional soccer players, but if the athlete participates in an event that is inherently moderate intensity, a 10-week antioxidative supplementation might have a larger effect on plasma SOD than a 10-week placebo intake (Margaritis et al., 2003). However, the practical application reference value of this speculation should be considered more carefully. There might be a more efficient anti-oxidative supplementation that hasn’t been found. Moreover, professional athletes do not often need to participate in physical exercise tests, the primary purpose of their daily training is to maintain their performances. Therefore, it should not simply be assumed that anti-oxidative supplementation doesn’t have extra value for professional athletes.

Third, according to the result of pair-wise meta-analysis and the meta-regression, it could be concluded that, for the oxidation and anti-oxidation balance under resting-state, anti-oxidative supplementation might be beneficial for most adults, but the effect size would largely depend on their daily life activities. Moreover, long-term anti-oxidative supplementation and long-term physical exercise might not create a simple superposition effect, on the contrary, they may even affect each other’s positive effects. The difference might come from the heterogeneity within the protocols such as the type of anti-oxidative supplementation, the intensity of physical exercise, and the overall health status of the participants. This speculation has been confirmed by many other studies. For example, according to the result of the three-armed trial made by Carrera-Quintanar in 2015 (Carrera-Quintanar et al., 2015), which is that a long-term intake of vitamin C and E might have a larger effect than a long-term intake of both vitamin C and E and Lippia, and a long-term intake of Lippia might have a negative effect on plasma SOD. Nevertheless, Jowko’s research in 2012 found that an acute intake of 640mg green tea polyphenols would not attenuate exercise-induced oxidative stress and muscle damage (Jówko et al., 2012), Deminice’s work in 2013 showed that creatine supplementation would inhibit the increase of inflammation markers TNF-α and CRP, but not oxidative stress markers, caused by acute exercise (Deminice et al., 2013), the study of da Silva in 2019 found that β-alanine and sodium bicarbonate could increase the estimated glycolytic contribution to high-intensity intermittent exercise (da Silva et al., 2019), and the study of Goldfarb in 2005 demonstrated that vitamin E, C, and selenium supplementation could attenuate the rise in malondialdehyde, which was a marker of oxidation in the body (Goldfarb et al., 2005).

Four, when it comes to the body’s adaptation to oxidative stress from a one-time physical exercise, it must be emphasized at first that the result of the pair-wised meta-analysis of the different effects of supplementation and placebo intake both in long-term and one-time protocol could only be discussed under total population and generally active population, since the subgroup pair-wised meta-analysis in other three population only included one study. It would make the result confusing and unreliable. According to the results of the pair-wised meta-analysis, both in total population and generally active subgroup, a long-term intake of antioxidative supplementation has a small but statistically insignificant advantage than placebo, whereas a one-time intake of antioxidative supplementation seemed to have a statistically insignificant disadvantage over placebo. The discrepancy indicated that only using anti-oxidative supplementation without changing lifestyle might not be the right choice to optimize the oxidation and anti-oxidation balance of the body. However, as mentioned above, the results should be interpreted with caution because that they came from one study. A randomized controlled trial found that results of this study indicated that the exact antioxidant effects of Artichoke extract in athletes were still not clarified (Atashak et al., 2019), the result of another randomized trial showed that short-term anti-oxidative supplementation with dosages of 1200 and 2400 mg/day of garlic extract didn’t show any effects on indicators of oxidation and anti-oxidation balance in male football players (Jahangard et al., 2013), and Su’s study in 2008 suggested that the anti-inflammatory and anti-oxidative effects of allicin were still unclear. More research is needed for a comprehensive conclusion in this perspective in the future.

Last but not the least, the results of the meta-analysis need to be interpreted with caution. On one hand, there is no official standard to evaluate and assess the overall oxidation and anti-oxidation balance in the human body because that the oxidative and anti-oxidative procedure in the human body is extremely complex and the measurement methods of each indicator are various with different precision and scales. On the other hand, the body’s adaption to oxidative stress and its overall health would also be influenced by other factors such as psychological status and environment. Although this review is based on the hypothesis that the tend to antioxidant direction of oxidation and anti-oxidation balance is positive for health, it should not be forgotten that the overall health is complex, there are many dynamic balances in the human body, and the status of one dynamic balance could not represent the overall health. For example, antioxidative supplementation might raise the overall anti-oxidative capacity while to some extent suppressing the body’s anti-oxidative defenses. Therefore, a reduction in plasma SOD in a certain situation might not be a negative signal.

The primary limitation of this review would be that, as the primary outcome of this review, the plasma SOD is the only one superoxide dismutase that could be found outside cells from the three different isoforms of SOD, and most exercise studies didn’t measure this specific isoform. It means that the plasma SOD’s bio-plausibility of its role in exercise human studies is still unclear. The most measurement of SOD in human exercise studies is the SOD in blood cells and muscle cells such as RBCs and leukocytes. This limitation might induce a public bias in this systematic review and meta-analysis and bring confusion to this review. Further research should try to reveal the evidence for major redundancy in the biological roles of EC-SOD and other isoforms of SOD.

Another important limitation of this systematic review is the statistical power of some pair-wise meta-analyses. Due to limited included evidence, the result of some subgroups would have low statistical power. For example, there were 6 pair-wise meta-analyses of different physical exercise and supplementation protocols for increasing plasma SOD under resting state, however, all of these pair-wise meta-analyses only included one trial. Nevertheless, according to the advantage of network meta-analysis, low statistical power doesn’t mean an incredible conclusion. It should be emphasized that in network meta-analysis, which is based on the Bayes’ theorem, each evidence could affect the rank of probability. Therefore, once evidence meet included criteria, it should not be excluded due to its low statistical power. What only could do is treated these results with caution and more research is needed in the future.

## Conclusion

There are two main conclusions of this systematic review. One conclusion is that maintaining a regular physical exercise routine would be beneficial to the body’s anti-oxidative potential, the intensity of the exercise should be set according to the daily life activity of the individual. The optimal exercise intensity is positively correlated with an individual’s daily life activity. For physically inactive persons, it is recommended to increase their daily life activities at first. The other conclusion is that anti-oxidative supplementation could have some positive effects on the body’s anti-oxidative status, but the effect is limited and probably has some pre-conditions. At the same time, anti-oxidative supplementation might have a complex interaction with physical exercise. It should be careful to set the combination of physical exercise and anti-oxidative supplementation, and more research is needed to explore the best combination protocol. Therefore, it is recommended to keep a regular physical exercise routine and gradually increase its intensity to maintain and enhancing the body’s antioxidation potential, and it is not recommended to only use anti-oxidative supplementation without changing lifestyle to optimize the oxidation and anti-oxidation balance of the body.

## Author Contributions

YX and ML: conceptualization and writing—review and editing. YX, ML, and UU: methodology and investigation. YX, UU, and YG: formal analysis. GF and YG: resources. YX and UU: data curation. UU, GF, and YG: supervision. All authors have read and agreed to the published version of the manuscript.

## Conflict of Interest

The authors declare that the research was conducted in the absence of any commercial or financial relationships that could be construed as a potential conflict of interest.

## Publisher’s Note

All claims expressed in this article are solely those of the authors and do not necessarily represent those of their affiliated organizations, or those of the publisher, the editors and the reviewers. Any product that may be evaluated in this article, or claim that may be made by its manufacturer, is not guaranteed or endorsed by the publisher.

## Footnotes

1
http://drugis.org/software/addis/index
