# Supplementary material for: Effect of Physical Exercise Under Different Intensity and Antioxidative Supplementation for Plasma Superoxide Dismutase in Healthy Adults: Systematic Review and Network Meta-Analysis
Source: Front Physiol. 2022 Feb 3;13:707176. doi: 10.3389/fphys.2022.707176 (PMC8850976; doi:10.3389/fphys.2022.707176)
Supplement: Supplementary file 1 [file Image_1.pdf]

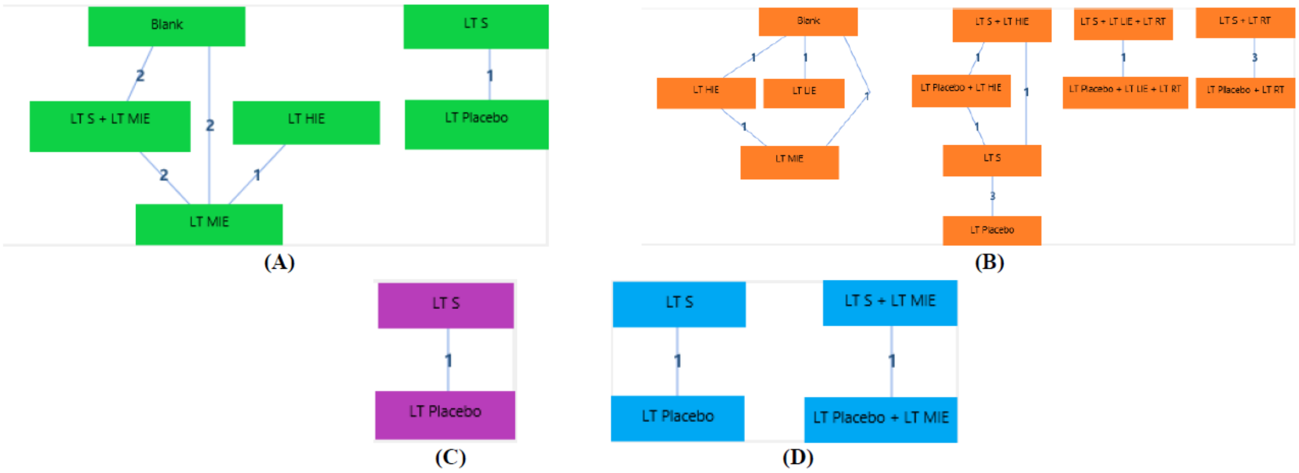

Supplementary Figure 1. The network geometry of different physical exercise and supplementation protocols for increasing plasma SOD under resting state. (A) Physically inactive population; (B) Generally physically active population; (C) Physically active population; (D) Professional athletes. (LT: long-term; S: supplementation; LIE: low-intensity exercise; MIE: moderate-intensity exercise; HIE: high-intensity exercise; RT: resistance training).
