# Supplementary material for: Effect of Physical Exercise Under Different Intensity and Antioxidative Supplementation for Plasma Superoxide Dismutase in Healthy Adults: Systematic Review and Network Meta-Analysis
Source: Front Physiol. 2022 Feb 3;13:707176. doi: 10.3389/fphys.2022.707176 (PMC8850976; doi:10.3389/fphys.2022.707176)
Supplement: Supplementary file 2 [file Image_2.pdf]

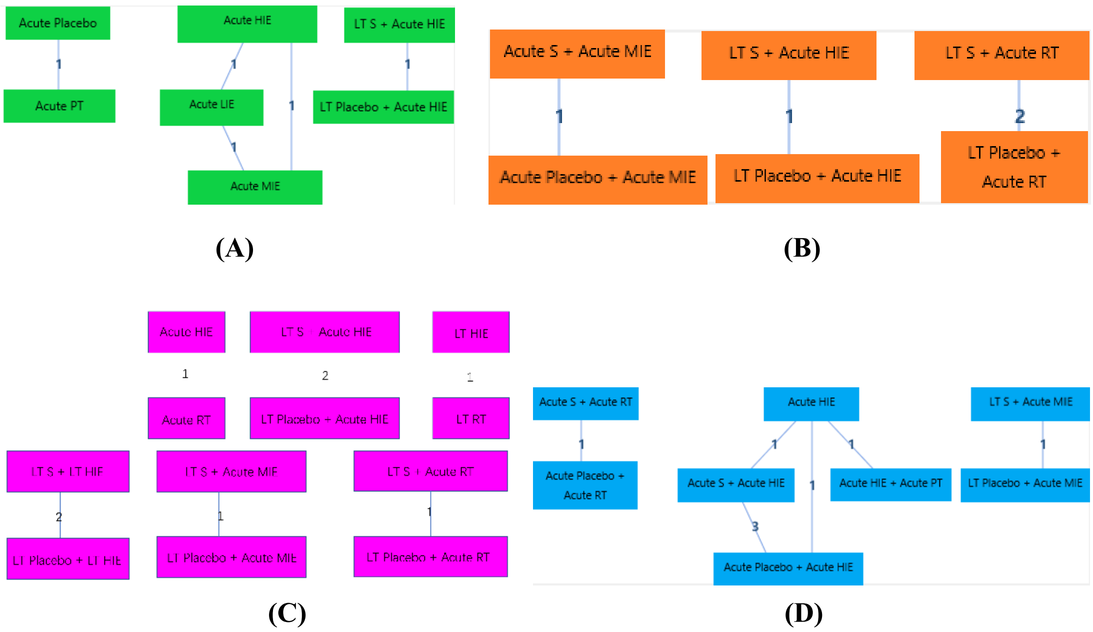


**Supplementary Figure 2. The network geometry of different supplementation and physical exercise protocols on the increasing plasma SOD after exercise tests. (A) Physically inactive population; (B) Generally physically active population; (C) Physically active population; (D) Professional athletes. (LT: long-term; S: supplementation; LIE: low-intensity exercise; MIE: moderate-intensity exercise; HIE: high-intensity exercise; RT: resistance training).**
